# Experimental observation of topological Dirac vortex mode in terahertz photonic crystal fibers

**DOI:** 10.1038/s41377-026-02197-6

**Published:** 2026-01-30

**Authors:** Hongyang Xing, Zhanqiang Xue, Perry Ping Shum, Longqing Cong

**Affiliations:** 1 State Key Laboratory of Optical Fiber and Cable Manufacture Technology, Department of Electrical and Electronic Engineering, Southern University of Science and Technology, Shenzhen, China; 2 Guangdong Key Laboratory of Integrated Optoelectronics Intellisense, Southern University of Science and Technology, Shenzhen, China

**Keywords:** Photonic crystals, Terahertz optics

## Abstract

Photonic crystal fibers have significantly advanced optoelectronics, enabling a wide range of applications from communications to sensing and imaging. A long-standing challenge in these areas has been achieving pure single-polarization single-mode (SPSM) waveguiding for high-quality information transmission. Traditional approaches, however, inevitably introduce polarization dispersion and operate within a narrow bandwidth. Recent advancements in topological phases offer a promising opportunity to access previously unattainable mode properties, though experimental demonstrations remain scarce. In this work, we present the first experimental observation of a topologically protected photonic Dirac vortex mode that supports pure SPSM propagation in terahertz fibers. Utilizing terahertz scanning near-field microscopic spectroscopy, we map the temporal, spectral, and spatial characteristics of the topological mode, providing insights into its mode profile, dispersion, effective area, and numerical aperture. We demonstrate a single linearly dispersed Dirac vortex mode with a single vortex polarization and a broad 85.7% fractional bandwidth. This breakthrough fills a crucial gap in the development of SPSM fibers and introduces a comprehensive methodology for exploring mode properties, paving the way for advancements in terahertz optoelectronics, topological photonics, and specialty optical fibers.

## Abstract (web summary)

We report the first experimental demonstration of a topological photonic Dirac vortex mode in terahertz fiber, enabling unprecedented broadband single-mode single-polarization transmission.

## Graphical abstract

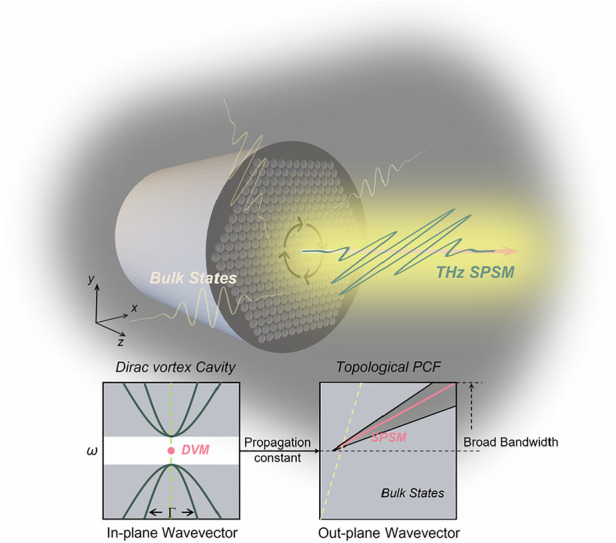

## Introduction

**Fig. 1:**
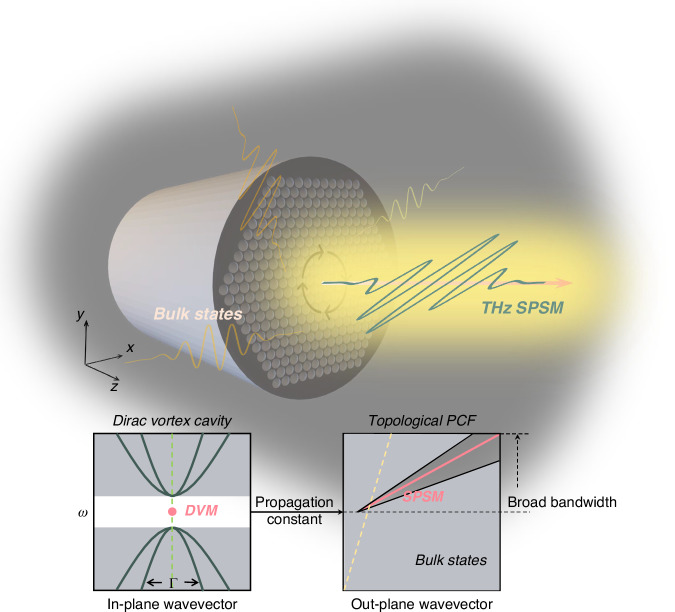
Schematic diagram of DVM in the PCF. The DVM is transformed into a propagating single mode with vortex polarization possessing a nonzero out-plane wavevector

Topologically protected photonic Dirac vortex modes (DVMs) reside in the mid-gap region and exhibit full spectral isolation from bulk states throughout the Brillouin zone in topological photonic vortex cavities with Kekulé phase modulation and disclination cavities^1–11^. These photonic DVMs are fundamentally linked to the mid-gap Jackiw-Rossi zero-energy states^12,13^, which solve the 2D Dirac equation with a topologically protected vortical mass term. Recent implementations of photonic DVMs in topological vortex cavities have enabled lasing functionality^14–17^. Significantly, introducing a nonzero out-of-plane wavevector component (*k*_z_ ≠ 0) triggers a transformation of DVMs from localized to propagating modes (Fig. 1, see Supplementary Section 15), a phenomenon theoretically predicted in photonic crystal fibers (PCFs) that maintain single-polarization single-mode (SPSM) operation^18^.

SPSM characteristic proves particularly advantageous in communication systems by effectively suppressing mode and polarization-dependent crosstalk. PCF-based SPSM implementations have demonstrated superior performance owing to their unique structure, guiding mechanism, and tunable optical properties^19–23^. High birefringence remains a prevalent strategy for realizing SPSM fibers, typically achieved either by engineering fundamental mode cutoff frequencies or suppressing undesired modes through selective attenuation. Established implementations exploit structural asymmetry^24,25^, index matching coupling technique^26^, anisotropic materials^27,28^, and anti-resonant fiber architectures^29–31^ to induce such birefringence. These methods, however, inherently sustain two non-degenerate polarization states, leading to residual modal coupling and polarization-mode dispersion that degrade signal integrity through pulse broadening and polarization crosstalk. Furthermore, the reliance on cutoff-frequency-based mode selection fundamentally restricts the operational bandwidth of SPSM fibers.

In next-generation wireless networks, terahertz (THz) waves emerge as a cornerstone technology offering unparalleled advantages in transmission capacity, sub-millisecond latency, and massive device connectivity. Nevertheless, free-space THz propagation faces inherent limitations involving pronounced atmospheric attenuation, which severely constrains effective communication distances. Significant progress has been made in utilizing traditional photonic crystal line defect waveguides^32^ and topological valley-Hall photonic crystals^33–35^ for THz wave guided transmission. Besides, waveguided THz transmission through specially designed fibers presents a viable strategy to overcome these challenges, and precise control over modal characteristics and polarization states with SPSM properties is essential for nondestructive imaging, sensing, and communications.

In this work, we experimentally demonstrate the pure single-polarization, single-mode characteristics of DVMs in PCFs using time-resolved terahertz scanning near-field microscopic spectroscopy (THz-SNMS). Unlike conventional time-domain spectroscopy (THz-TDS), THz-SNMS enables direct spatial mapping of mode profiles while integrating spectrally resolved characterization capabilities^36^. By incorporating the short-time Fourier transform (STFT) algorithm, we reconstruct the mode dispersion, propagation dynamics, effective area, and numerical aperture, collectively revealing a linearly dispersed DVM with an extensive 85.7% fractional bandwidth. Additionally, we estimate the low confinement loss of the DVM, an intrinsic property at the Γ point in the absence of an in-plane wavevector, where all bulk states undergo exponential decay into free space. The single-vortex polarization state is further confirmed through the observed electric field vector distributions.

## Results

### Theoretical realization

**Fig. 2:**
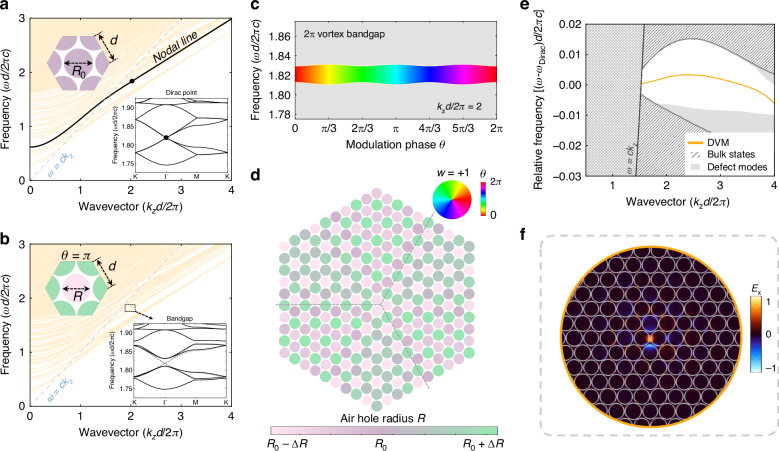
Topological PCF realized by Kekulé modulation. **a** Projected band structure of the primitive superlattice uniformly extended in the out-plane direction at different propagation constants. The side length *d* is 1.82 mm, and the nodal line, marked by the black solid line, consists of Dirac points in the inset 2D band structures. **b** Projected band structure of the Kekulé modulation superlattice with Δ*R* = 0.02 *d* and *θ* = *π*. The nodal line is lifted, forming a complete bandgap due to broken inversion symmetry. The blue dashed line represents the light line. Inset: lattice structures and in-plane band structures at *k*_z_*d*/2π = 2. **c** 2π vortex bandgap obtained by modulating *θ* at *k*_z_*d*/2π = 2. The rainbow color represents the modulation phase. **d** Modulation of air-hole radii in the photonic structure, varying from *R*_0_ − Δ*R* to *R*_0_ + Δ*R*, and *R*_0_ = 0.46 *d* and Δ*R* = 0.02 *d*. **e** The band structure of the PCF near the bandgap where DVM exists. **f** Mode profiles of the normalized *x*-polarized electric fields for DVM

A photonic crystal with a hexagonal superlattice structure is considered, consisting of air holes drilled into a host medium, where the period is *d* and the radius of identical air holes is *R*_0_ = 0.46 *d* (see Supplementary Section 13). A quadra-degenerate Dirac point arises due to in-plane inversion symmetry and band folding at *Γ* point in the Brillouin zone (inset in Fig. 2a at *k*_z_*d*/2*π* = 2, see Supplementary Section 1). Projection of the two-dimensional band diagram at varied propagation constants (*k*_z_) assembles a nodal line with a collection of Dirac points. The nodal line can be transformed into a complete bandgap by applying a Kekulé superlattice modulation, where the radii of the air holes comply with$R={R}_{0}+\varDelta R\,\cos ({{\bf{K}}}_{\mathrm{int}}\cdot {{\bf{r}}}_{0}+\theta )$. Here, Δ*R* and *θ* represent the Kekulé modulation amplitude and phase, respectively. ${{\bf{K}}}_{\mathrm{int}}=K-K^{\prime} =(8\pi /3d,0)$ implies the inter-valley coupling between *K* and $K^{\prime}$ valleys, and **r**_0_ is the coordinate position relative to the lattice center. At $\theta =\pi$, the nodal-line-gapped band structure is shown in Fig. 2b, and a complete bandgap opens, as shown in the inset.

Interestingly, the bandgap acquires a 2π vortex through continuous tuning of the phase *θ*, as illustrated in Fig. 2c at *k*_z_*d*/2*π* = 2. A topological phase transition occurs at $\theta =\pi$, shifting the system from a topologically nontrivial to a trivial state (see Supplementary Section 1). To induce this transition, we apply a vortex Kekulé phase modulation by introducing a position-dependent air hole radius distribution (Fig. 2d)

1 $$R={R}_{0}+\Delta R\,\cos [{{\bf{K}}}_{\mathrm{int}}\cdot ({\bf{r}}-{{\bf{r}}}_{0})+\theta ({\bf{r}})]$$

where ${\bf{r}}(x,y)$ denotes the position of the hole center, $\theta ({\bf{r}})=w{\tan }^{-1}(y/x)$ is a position-dependent modulation phase with winding number *w* = +1, and **r**_0_ is a *w*-dependent core center conserving a C_3_ symmetry (see Supplementary Section 2 and Section 21). As a result of the topological phase transition, a topologically protected defect mode, identified as the DVM in the PCF, emerges (see Supplementary Section 17). This mode is spectrally localized within the bandgap and spatially confined at the core, as shown in Fig. 2e, f. Additionally, the field remains localized around the fiber center along the propagating direction (see Supplementary Section 14). At the edge of the bandgap, local-defect modes appear as a result of noncontinuous variation of geometric parameters (grey in Fig. 2e, see Supplementary Section 3), which will not exert impact on the DVM.

### Experimental observation of DVM

**Fig. 3:**
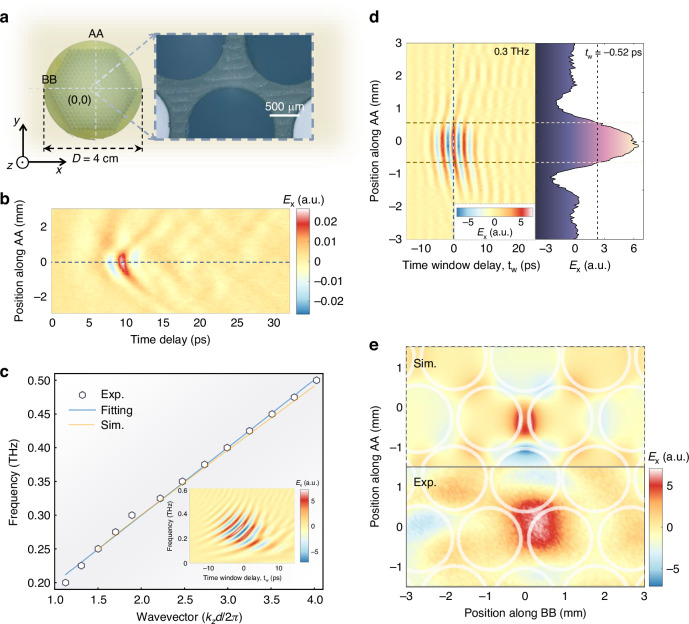
Experimental characterization of DVM. **a** Microscopic images of the 3D-printing PCF cross-section. The coordinate system is defined based on symmetry with the origin at the center of the PCF. Scanning paths along AA and BB are illustrated. **b** Measured time-domain *x*-polarized electric field distribution along path AA. **c** Experimentally reconstructed band structure of the DVM with a corresponding fitting line. The linear band structure exhibits excellent agreement with simulation results. Inset: temporal-spectral distributions obtained by performing STFT at the origin along the dashed path in (**b**). **d** Temporal-spatial distributions at 0.3 THz along path AA. The right panel shows the spatial electric field distribution at *t*_w_ = −0.53 ps, with the dashed line indicating the position where *E*_x_ drops to *e*^-1^ of its maximum value. **e** Spatial distributions of the DVM at 0.3 THz

The properties of the DVM are experimentally investigated by fabricating a PCF using high-temperature resin with a refractive index 1.631 + *i*0.009 in the THz regime via 3D printing (see Methods). The cross-sectional structure, shown in Fig. 3a, features air holes arranged according to the Kekulé modulation. The spatial profile of the DVM is characterized using THz-SNMS (see Methods), wherein near-field information is captured by a THz microprobe with spatial (10 μm) and spectral (0.1–1.5 THz) resolution across the fiber cross-section. Orthogonal scanning paths are defined along AA and BB as illustrated in Fig. 3a.

Figure 3b presents the directly measured *x*-polarized time-domain signals along path AA, excited by a focused THz pulse from the opposite fiber end. The results reveal strong energy confinement around the core. To further analyze the spatial, spectral, and temporal characteristics, near-field distributions are reconstructed using the STFT, where a sliding time window decomposes the temporal signal into a two-dimensional time-frequency representation (see Supplementary Section 4). The inset of Fig. 3c illustrates the spectral dynamics, revealing a series of propagating dispersion relations for the DVM derived from STFT analysis of the time-domain distributions. The phase evolution of the dispersion appears as a series of striations, resembling the propagation of a dynamic band structure. The dispersion relation of the DVM is indirectly determined by analyzing the dynamic phase evolution of its frequency-dependent modal components (see Supplementary Section 5). As evidenced in Fig. 3c, the experimentally reconstructed band structure closely agrees with numerical results. Additionally, the phase velocity exhibits a consistent trend between experimental observations and theoretical predictions, where higher (lower) frequencies correspond to slower (faster) phase velocities (see Supplementary Section 6).

Temporal-spatial distributions reveal the dynamic evolution of the DVM as shown in Fig. 3d (at 0.3 THz as an example, see Supplementary Section 5). The mode is tightly confined around the origin, with a decay length of ~1 mm, as observed from the electric field along path AA at *t*_w_ = −0.53 ps (Fig. 3d). By incorporating field distributions along path BB, complete 2D mode patterns are reconstructed (Fig. 3e at 0.3 THz, and see Supplementary Sections 7 and 8). The experimentally observed mode patterns closely match theoretical predictions, with only a slight expansion in the measured distribution, which can be attributed to the half-wavelength propagating divergence between the PCF cross-section and the microprobe during measurements.

### Performance characterization

**Fig. 4:**
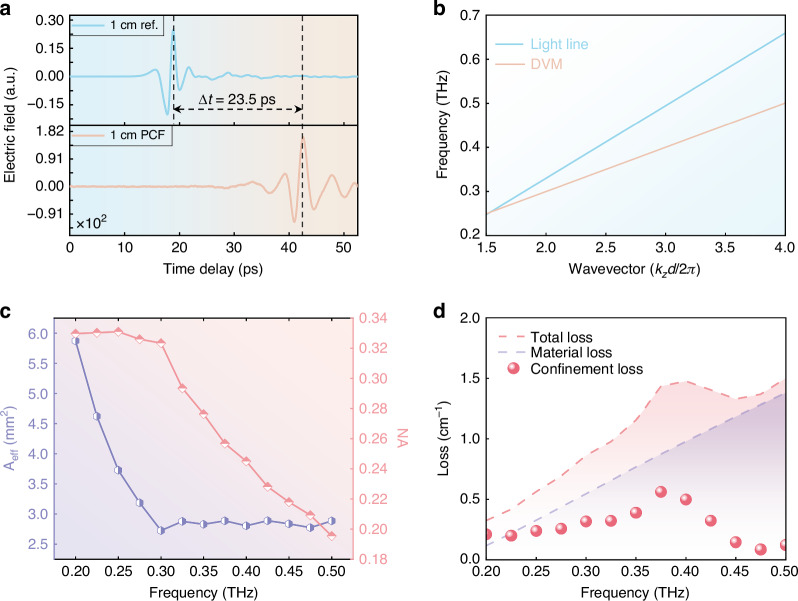
Performance characterization. **a** The group delay of the DVM is determined by measuring the time delay between pulses propagating through an equal distance (1 cm) in air and the PCF. **b** DVM dispersion extracted from the data in Fig. 3c, exhibiting a perfectly linear dispersion and a group velocity of 0.5848*c* as compared with the light line in free space. **c** Effective area for the mode confinement and numerical aperture. **d** Total loss, material loss, and confinement loss

The DVM dispersion is extracted from the measured temporal-spectral distributions, exhibiting an exceptionally linear relationship akin to the light line, with a group velocity of 0.5848*c*. This dispersion is characterized by directly measuring the time delay of THz pulses propagating through both air and the PCF over an identical distance of 1 cm. During measurements, the microprobe is precisely positioned at the mode maximum. As shown in Fig. 4a, the group delay of pulse peaks is determined to be 23.5 ps, aligning well with the estimation based on group velocity (Fig. 4b). The overall pulse shape remains preserved due to the linear dispersion, with slight broadening attributed to the narrower bandwidth of the DVM compared to that of the incident THz pulse.

The spatial confinement of the DVM is characterized by its effective area (A_eff_). The effective mode area is calculated from the electric field distribution as

2 $${A}_{eff}=\frac{(\iint {|{E}_{x}(x,y)|}^{2}dxdy{)}^{2}}{\iint {|{E}_{x}(x,y)|}^{4}dxdy}$$

where the integral is taken over the PCF cross-section. As shown in Fig. 4c, the effective mode area rapidly decreases to 2.7 mm^2^ within the DVM band from 0.3 to 0.5 THz. This value is two orders of magnitude smaller than state-of-the-art PCFs^37–41^, occupying only 0.0537% of the total cross-sectional area (Table 1). Such extreme confinement facilitates the dense integration of functional components in chip-scale THz photonic systems. The strong mode confinement also results in a large numerical aperture (NA), estimated using the relation^42^

3 $$NA={(1+\pi {A}_{eff}/{\lambda }^{2})}^{-1/2}$$

**Table 1:** Effective mode area compared with the state-of-the-art THz PCFs

Year	Frequency (THz)	Relative A_eff_ (A_eff_/cross-section, %)	Experimental result
2018^38^	1	5.56	No
2023^37^	0.8	2.785	No
2023^39^	1.8	2.4	No
2023^40^	1.9	3.146	No
2025^41^	2.2	2.948	No
This work	0.3	0.0537	Yes

At 0.3 THz, the NA is approximately 0.32 as shown in Fig. 4c, which falls within the conventional range for THz fibers (NA: 0.2–0.5)^43–45^.

In terms of the total losses (*α*_*tot*_), we separately evaluate the material loss (*α*_*mat*_) and the confinement loss (*α*_*con*_ = *α*_*tot*_ − *α*_*mat*_). The total loss is determined using the cutback measurement technique, in which transmission signals are measured from two PCFs of different lengths. The difference in transmission accounts for the total losses induced by length variation while excluding coupling attenuation (see Supplementary Sections 9 and 18). The frequency-dependent total losses of the DVM are presented in Fig. 4d. By utilizing the imaginary part of the effective refractive index of the material (around 0.004 at 0.3 THz, see Supplementary Section 10), the confinement loss of the DVM is extracted, revealing a minimum value of 0.198 cm^−1^ at 0.225 THz. The overall confinement loss remains around 0.275 cm^−1^ in the 0.2–0.375 THz range. Beyond 0.4 THz, significant fluctuations appear in the confinement loss values, which are attributed to the degraded signal-to-noise ratio of the DVM under high-frequency excitation (see Supplementary Section 9).

### Vortex polarization

**Fig. 5:**
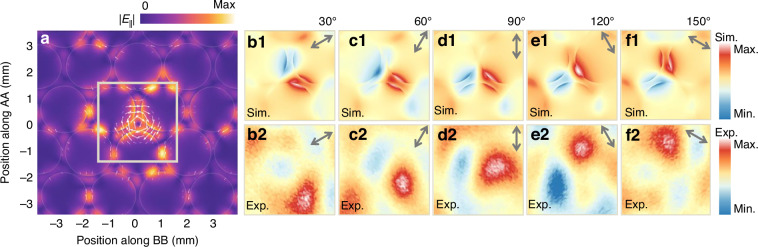
Vortex polarization. **a** Simulated electric field vector distribution of the DVM mode, indicating a vortex property. **b1–f1** Simulated mode patterns excited by linear polarization at varied angles at 0.3 THz within the region marked by the square in (**a**). **b2–f2** Corresponding experimental demonstration of the DVM mode patterns

Another key characteristic of the DVM is its vector electric field distribution, which exhibits a vortex-like polarization governed by the Kekulé modulation of the vortex phase, as illustrated in Fig. 5a. To experimentally verify this vector polarization, we analyze the mode pattern under excitation by a linearly polarized incident wave with varying orientations. A dipole-like mode pattern emerges, with the nodal line consistently aligning parallel to the incident linear polarization vector, as confirmed by simulations in Fig. 5b1–f1. In THz-SNMS experiments, the same field patterns are observed (Fig. 5b2–f2), with the nodal lines rotating in accordance with the orientation of the incident linear polarization vector at 0.3 THz. This direct experimental evidence unambiguously confirms the vortex polarization property of the DVM and polarization alignment-free (see Supplementary Section 20) SPSM operation for the THz PCF.

## Discussion

DVMs exhibit core photonic properties essential for fiber applications: pure SPSM operation, broad bandwidth, linear dispersion, and small mode volume. We fabricated Kekulé-phase-modulated photonic crystal fibers via 3D printing, experimentally demonstrating 85.7% bandwidth SPSM operation with a mode area occupying merely 0.0537% of the fiber cross-section, as characterized by time-resolved THz-SNMS. In the current design, mode propagation is primarily limited by material absorption in the polymer components. This limitation could be overcome by employing low-loss THz materials, such as methyl pentene copolymer (TPX) and polytetrafluoroethylene (PTFE), which exhibit near-zero absorption below 0.5 THz, in combination with high-precision femtosecond laser direct writing techniques^46,47^. Theoretically, materials with zero absorption coefficients combined with flawless fabrication techniques would minimize the confinement losses of the SPSM PCF to negligible levels, thereby significantly enhancing transmission distance (see Supplementary Section 11). Notably, DVMs completely eliminate polarization-mode dispersion inherent in conventional PCFs by exclusively sustaining vortex-polarized topological states from 0.2 THz to 0.5 THz, achieving the broadest operational bandwidth reported to date (Table 2).

**Table 2:** Comparison with the state-of-the-art THz SPSM fibers

Platforms	Conceptual advancement	Birefringence	Polarization dispersion	Confinement loss (α_con_)	Frequency range (Fractional bandwidth^a^)
Hollow waveguide^24^	Asymmetry	Yes	Yes	1.35 × 10^−2^ cm^−1^–1.38 × 10^-2^ cm^−1^	0.06–0.14 THz (80%)
PCF^26^	Index-matching coupling	Yes	Yes	~1.06 × 10^−2^ cm^−1^	1.575–1.8 THz (13.3%)
PCF^25^	Asymmetry	Yes	Yes	Not considered	0.84–1.16 THz (32%)
PCF^28^	ENZ material	Yes	Yes	≤3.16 cm^−1^	0.82–1.14 THz (32.7%)
PCF^27^	Asymmetry, ENZ and gain material	Yes	Yes	≤3.16 cm^−1^	1.10–1.74 THz (47.2%)
Hollow core fiber^31^	Anti-resonant fiber	Yes	Yes	1.12 × 10^−2^ cm^−1^	0.484–0.517 THz (33%)
Hollow core fiber^51^	Anti-resonant fiber	Yes	Yes	≤1.059 × 10^−2 ^cm^−1^	0.82–1.24 THz (20.4%)
Negative curvature fiber^52^	Asymmetry cladding	Yes	Yes	~1.001 × 10^−2^ cm^−1^	0.98–1.02 THz (4%)
PCF^53^	ENZ material	Yes	Yes	Not considered	1.3–1.5 THz (14.3%)
This work	DVM	No	No	~0.275 cm^−1^	0.2–0.5 THz (85.7%)

^a^Fractional bandwidth spans over central frequency

By incorporating the flexibility of PCFs, we foresee even more unique mode properties for specific applications, such as extreme mode volume through high-order topological corner states^48^ or Moiré lattice^49^, and reduced confinement loss with miniaturized BIC^50^. This advancement positions THz PCFs as pivotal components for integrated systems, enabling breakthroughs in quantum cascade laser architectures, sensing, distributed quantum networks, and sub-wavelength resolution imaging.

## Methods

### Fabrications and materials

The photonic crystal fiber (PCF) was fabricated using stereolithography-based 3D printing technology (Form 3+ from Formlabs), which provides a laser spot size of 85 μm and an axis resolution of 25 μm. For the printing material, we selected high-temperature resin due to its relatively low absorption in the THz regime, with a refractive index of 1.631 + *i*0.009 (see Supplementary Section 19). This material offers several benefits, such as excellent mechanical properties, with a post-cured temperature tolerance of up to 238 °C at 0.45 MPa and an ultimate tensile strength of 49 MPa, ensuring the structural integrity and durability of the fabricated fibers. Two prototypes of 1 cm and 2 cm lengths were fabricated to characterize the near-field profiles and total loss, respectively. The 3D printing process, while highly precise, does have inherent limitations, such as potential deformation of the air holes during the curing process and residual material left in the holes. These challenges were addressed through post-processing, including cleaning and precision machining. The material selection and the 3D printing process, while optimal for this study, are not without trade-offs, such as absorption losses and minor dimensional variations due to the fabrication process. However, the results demonstrate that these factors do not significantly impair the fiber’s performance in the THz regime (Supplementary Section 16).

### Measurements

THz-SNMS was used to measure the transmission field dynamics through the PCF, and the optical setup is shown in Supplementary Section 12. A femtosecond laser (800 nm, 38 fs, 80 MHz) was split into two coherent paths: pump and probe. The pump beam excites linearly polarized THz radiation from the photoconductive antenna. In the probe path, dispersion is compensated using a pair of gratings before the probe beam is coupled into an optical fiber, ensuring an output pulse width of 70 fs. A near-field photoconductive antenna, driven by the probe beam, covers a spectral range from 0.1 to 1.5 THz.

Time-domain terahertz pulses were obtained by moving the delay stage to adjust the difference between the pump and probe paths. Spatial information was acquired by performing a point-by-point scan in two dimensions (step size 50 µm, scan rate: 3 Hz). The DVM is effectively coupled by a focused THz beam to ensure sufficient overlap. During polarization measurements, the PCF was rotated relative to the polarization vector of incidence.

### Numerical method

Numerical simulations were carried out using finite element methods. A simplified 2D model of the fiber cross-section was modeled in the *xy*-plane. With continuous translational symmetry along the *z*-axis, an external out-of-plane wavevector was introduced into the model. The refractive indices of the resin and air were set to 1.631 + *i*0.009 and 1, respectively. In the simulations, a Floquet periodic boundary condition was applied to the unit cell of the periodic lattices, while a scattering boundary condition was applied to the cladding of the PCF with Kekulé modulation. The in-plane projected band diagrams are obtained by scanning the in-plane Bloch wavevectors while fixing the out-of-plane wavevector. Similarly, the out-plane projected band diagrams are constructed by calculating the eigenfrequencies corresponding to various out-of-plane wavevector values at high-symmetry points of the Brillouin zone and then assembling these data to generate the complete projected dispersion.

## Supplementary information

Supp Mater_Experimental observation of topological Dirac vortex mode in terahertz photonic crystal fibers

## Data Availability

The data are available from the corresponding author upon reasonable request.
