# Supplementary material for: Experimental observation of topological Dirac vortex mode in terahertz photonic crystal fibers
Source: Light Sci Appl. 2026 Jan 30;15:97. doi: 10.1038/s41377-026-02197-6 (PMC12859132; doi:10.1038/s41377-026-02197-6)
Supplement: Supplementary file 1 — Supp Mater_Experimental observation of topological Dirac vortex mode in terahertz photonic crystal fibers [file 41377_2026_2197_MOESM1_ESM.pdf]

Supplementary Material for Experimental observation of topological Dirac vortex mode in terahertz photonic crystal fibers

Hongyang Xing[1], Zhanqiang Xue[1], Perry Ping Shum[1,2], Longqing Cong[1,2*]

[1]*State Key Laboratory of Optical Fiber and Cable Manufacture Technology, Department of Electrical and Electronic Engineering, Southern University of Science and Technology, Shenzhen 518055, China.*

[2]*Guangdong Key Laboratory of Integrated Optoelectronics Intellisense, Southern University of Science and Technology, Shenzhen 518055, China.*

[*]conglq@sustech.edu.cn

Section 1. Topological phase transition in the Kekulé modulation.

Our model can be depicted as a graphene-like tight-binding model with out-plane direction uniform, as shown in Fig. S1a, which deduces to a two-valley Dirac Hamiltonian in the neighborhood of the in-plane Dirac point:

$$He^{-ik_z z} = \begin{bmatrix} 0 & t_1 & 0 & t_2 e^{i\vec{k}\cdot\vec{a_1}} & 0 & t_1 \\ t_1 & 0 & t_1 & 0 & t_2 e^{i\vec{k}\cdot\vec{a_2}} & 0 \\ 0 & t_1 & 0 & t_1 & 0 & t_2 e^{i\vec{k}\cdot\vec{a_3}} \\ t_2 e^{-i\vec{k}\cdot\vec{a_1}} & 0 & t_1 & 0 & t_1 & 0 \\ 0 & t_2 e^{-i\vec{k}\cdot\vec{a_2}} & 0 & t_1 & 0 & t_1 \\ t_1 & 0 & t_2 e^{-i\vec{k}\cdot\vec{a_3}} & 0 & t_1 & 0 \end{bmatrix} e^{-ik_z z} \qquad (S1)$$

where t_1 and t_2 signify the intra- and intercell couplings between adjacent lattice sites, respectively, while $\vec{k}$ denotes the in-plane wave vector. The lattice vectors $\vec{a_1}$, $\vec{a_2}$, and $\vec{a_3}$ are defined such that $\vec{a_3} = \vec{a_2} - \vec{a_1}$. The bulk band structures illustrated in Fig. S1b-d are derived from Eq. S1. When $t_1 = t_2$, the lattice arrangement facilitates wave propagation through a honeycomb channel, resulting in the emergence of two branches (valleys) of Dirac-cone dispersive states for the undisturbed band. Conversely, when t_1 differs from t_2, the introduction of a finite mass term facilitates intervalley coupling, leading to a bandgap at the Γ point. Notably, when the inter-supercell hopping is more than the intra-supercell hopping ($t_1 > t_2$), the system exhibits a topologically trivial

characteristic. However, when the inter-supercell hopping is less than the intra-supercell hopping ($t_1 < t_2$), a band inversion occurs, indicating a topological phase transition.

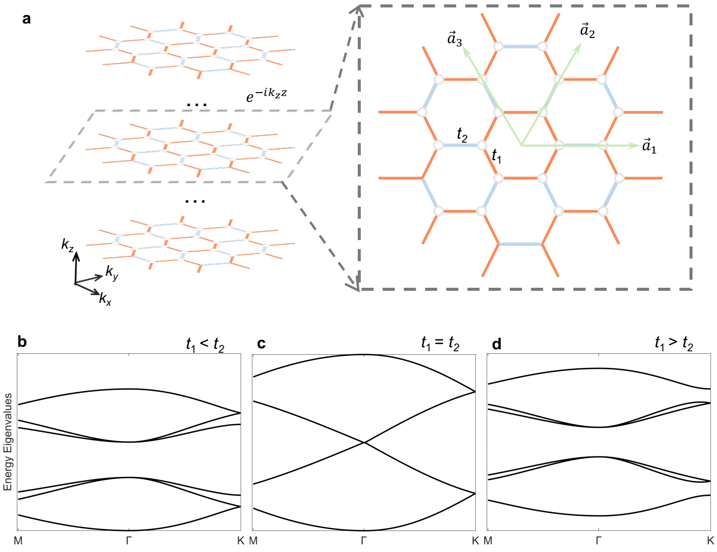

Fig. S1. a, Graphene-like tight-binding model. **b-d,** Band structure of the photonic lattice with different relative hopping amplitudes of t_1 and t_2.

Intervalley coupling is explored by altering the symmetry via a set of Kekulé modulation phases, which necessitates adjustments in the air hole radii in the designed lattice. The adjusted radii for each lattice site are described by $R = R_0 + \Delta R \cos(\mathbf{K}_{\text{int}} \cdot \mathbf{r}_0 + \theta)$, where $\mathbf{r}_0$ denotes lattice positions $(0,0)$, $(d,0)$, $\left(d/2, \sqrt{3}d/2\right)$, $\left(-d/2, \sqrt{3}d/2\right)$, $(-d,0)$, $\left(-d/2, -\sqrt{3}d/2\right)$, and $\left(d/2, -\sqrt{3}d/2\right)$. This radius change impacts the hopping amplitudes $t_{1,2}$ in the model by a magnitude $\delta t(\mathbf{r}_0)$. The formula for $R(\mathbf{r}_0)$ thus influences the real hopping values as

$$t(\boldsymbol{r}_0) = t_0 - \delta t(\boldsymbol{r}_0)\cos(\boldsymbol{K}_{int} \cdot \boldsymbol{r}_0 + \theta) = t_0 + \left[\Delta(\boldsymbol{r}_0)e^{i\boldsymbol{K}_{int}\cdot\boldsymbol{r}_0} + \Delta(\boldsymbol{r}_0)e^{-i\boldsymbol{K}_{int}\cdot\boldsymbol{r}_0}\right], (S2)$$

where t_0 defines the unperturbed hopping amplitude for the model and $\Delta(\boldsymbol{r}_0) = -\frac{1}{2}\delta t(\boldsymbol{r}_0)e^{i\theta}$ accounts for the band gap generation. The modulation phase θ predominantly governs the bandgap width due to intervalley coupling, as depicted in Fig. 3a in the main context. In addition, for a modulation phase $\theta = 0°$, the supercell

indicates a topological phase transition ($t_1 < t_2$), and for $\theta = 180°$, it aligns with a trivial phase ($t_1 > t_2$).

Additionally, our model has similar band diagrams to graphene-like tight-binding model. As illustrated in Fig. S2(a–c, left panels), we first consider the irreducible unit cell of the photonic crystal structure, whose Brillouin zone (BZ) is shown in Fig. R2(b, left panel). When the normalized out-of-plane wavevector satisfies $k_z d/2\pi$ = 2, the corresponding band structure (Fig. S2(c, left panel)) exhibits degenerate Dirac points located at the K_o and K_o' valleys.

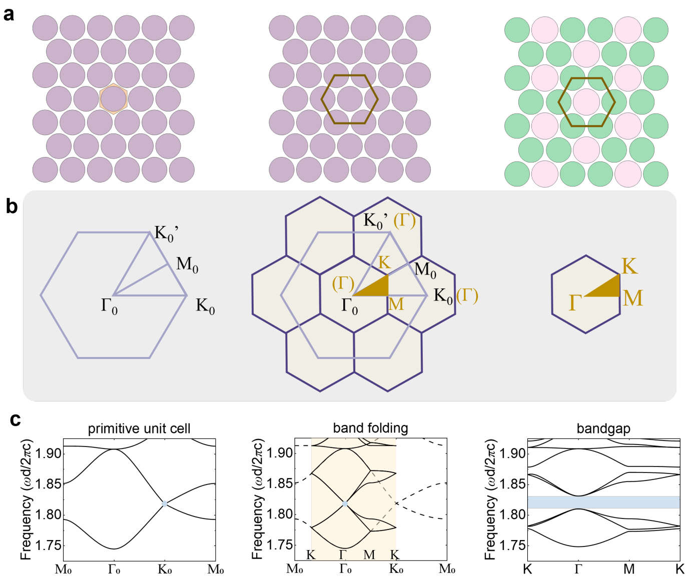

Fig. S2. The band folding process. a, The choices of unit cell for the photonic crystal structure. **b,** The corresponding BZs subjective to different unit cells. **c**, The band diagrams of different unit cells.

Next, we construct a supercell by expanding the periodicity of the unit cell, as depicted in Fig. S2(a, middle panel). The BZ of the supercell becomes 1/3 the size of the original one, i.e., $\Gamma M = (1/2)\Gamma_0 K_0$ and $\Gamma K = (2/3)\Gamma_0 M_0$. Consequently, the K_o and K_o' points of the primitive BZ are folded onto the Γ point of the supercell BZ. This folding process leads to the formation of a fourfold-degenerate Dirac point at Γ, as shown in Fig. R1-1(c, middle panel). When the supercell symmetry is subsequently broken through the Kekulé-type modulation introduced in our design, the fourfold degeneracy at Γ is lifted,

thereby opening a topological bandgap, as demonstrated in Fig. S2 (right panels).

Section 2. Core center conserving C_3 symmetry.

The Kekulé-modulated fiber cross-section structure requires a precise selection of the reference center coordinates and winding number (w) to satisfy C_3 rotational symmetry, as detailed in Fig. S3. In our design, the reference center is positioned at $(\frac{\sqrt{3}d}{6}, -\frac{d}{2})$ with a winding number $w = +1$.

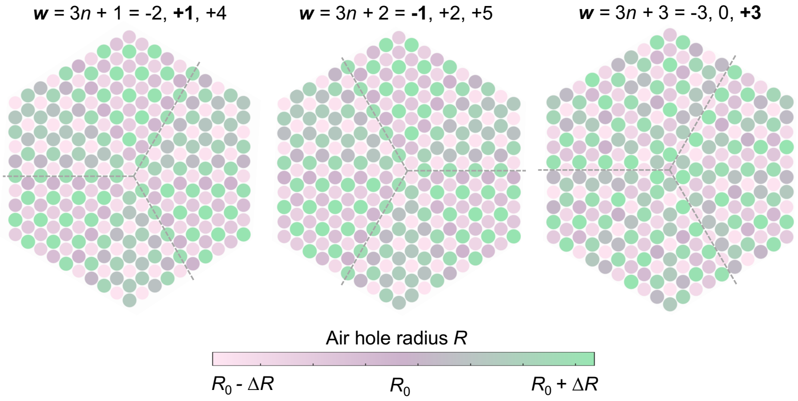

Fig. S3. Three types of PCF cross-sections conserving C_3 symmetry with different core positions. The cores of the three examples are set at $(\frac{\sqrt{3}d}{6}, -\frac{d}{2})$, $(-\frac{\sqrt{3}d}{6}, -\frac{d}{2})$ and $(0,0)$ with winding numbers of $w = +1$, $w = -1$ and $w = +3$, respectively.

Section 3. Local defect modes in the bandgap of the PCF.

Discrete variations in geometric parameters induce localized defect modes near the bandgap edges, constrained by the effective hole center coordinates, as demarcated in the grey-shaded region of Fig. 3c. This modal localization phenomenon arises from the interplay between structural disorder and the eigenfield distribution is shown in Fig. S4.

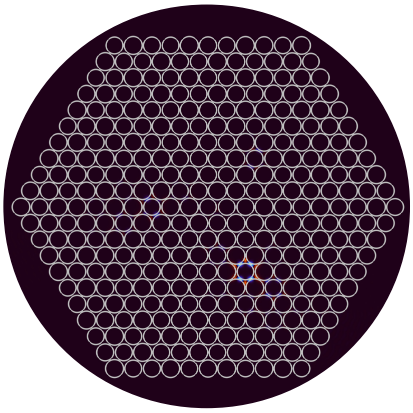

Fig. S4. An example of the local defect mode profile. These modes tend to arise near the bandgap and can be deemed as the extended modes of the bulk states.

Section 4. Short time Fourier transform (STFT).

As shown in Fig. S5, we use a hamming window as the time window function for the data processing of STFT as

$$w(t) = \begin{cases} 0.54 - 0.46\cos\left[\frac{2\pi(t - t_w)}{T}\right], t_w - \frac{T}{2} < t < t_w + \frac{T}{2}, \\ 0, otherwise \end{cases} \quad (S3)$$

where T is the full width of the window function. To optimize the tradeoff between the temporal and spectral resolutions, the window length was set to 3000 samples, with an overlap of 2999 samples between successive frames. The Fourier transform was performed with 10,000 points to enhance frequency resolution and accuracy in the spectral analysis.

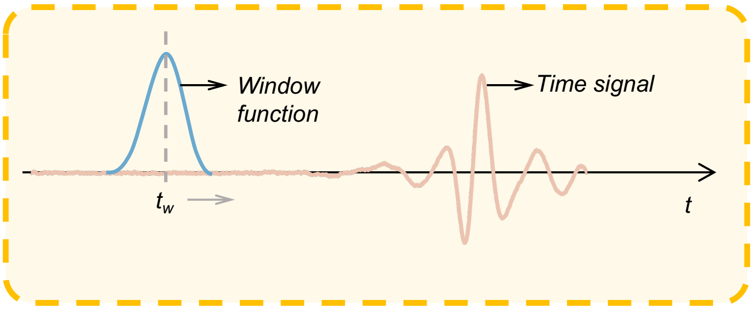

Fig. S5. Schematic diagram of STFT. STFT adds time-windowing to extract frequency information with localized time information, making it more suitable for dynamic frequency monitoring and time-frequency feature extraction of the single zero mode.

Section 5. Temporal-spatial information after short time Fourier transform.

Following the methodology in Fig. 3e, temporal-spectral analysis via STFT enables selective frequency component extraction from the measured distributions, as explicitly resolved in Fig. S6. Furthermore, the temporal dynamics of individual frequency components can be analyzed through position-specific temporal mode evolution, as illustrated in Fig. S7.

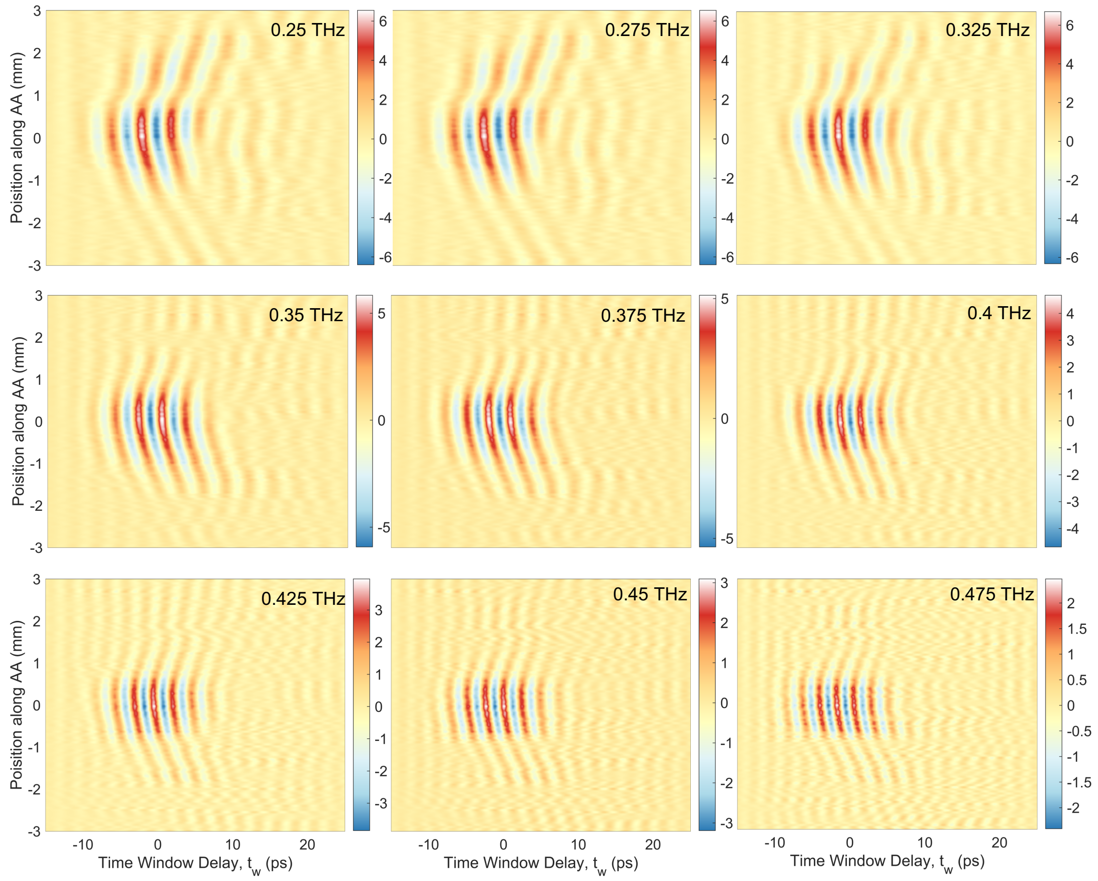

Fig. S6. Temporal-spatial maps along path AA at various frequencies.

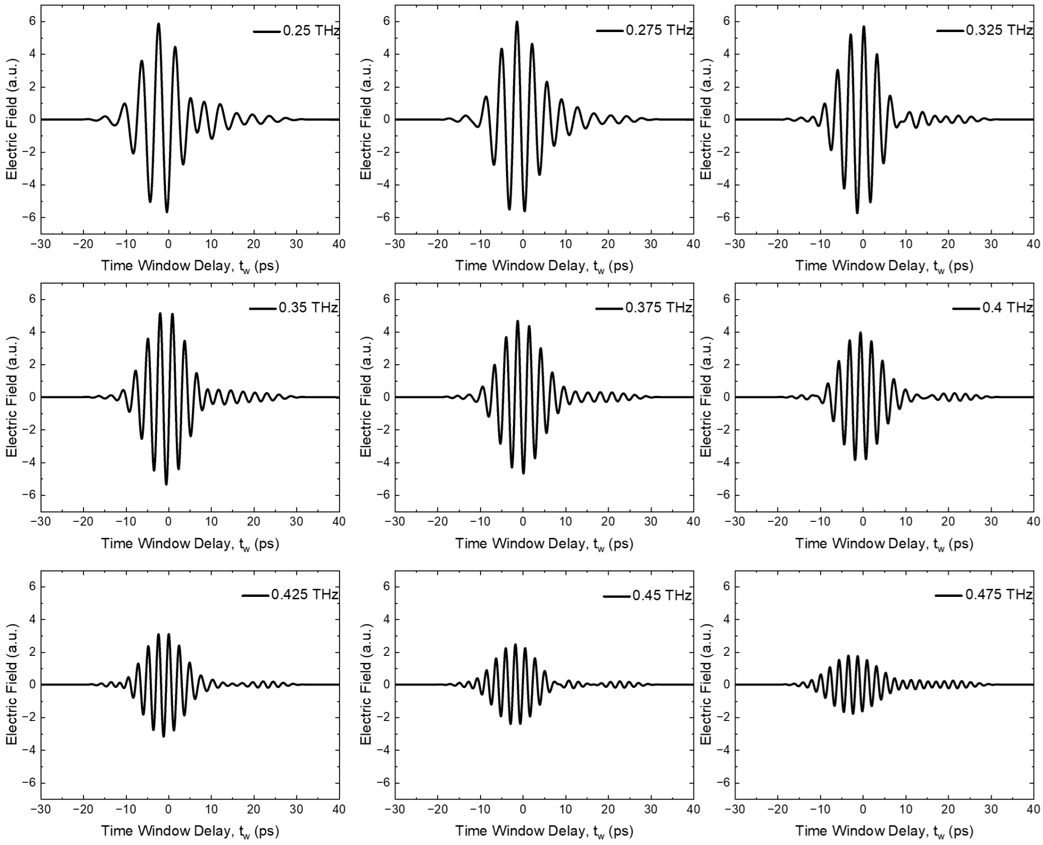

Fig. S7. Temporal dynamics of individual frequency components at the position of AA = 0.

Section 6. Phase velocities (v_p) at various frequencies.

By analyzing the temporal dynamics of individual DVM frequency components in Fig. S6, we extract their characteristic mode frequencies f_m. The phase velocities v_p at each frequency were then calculated through the relation $v_p = f_0 c/f_m$, where f_0 represents the operational carrier frequency and c implies the vacuum light speed, as depicted in Fig. S8.

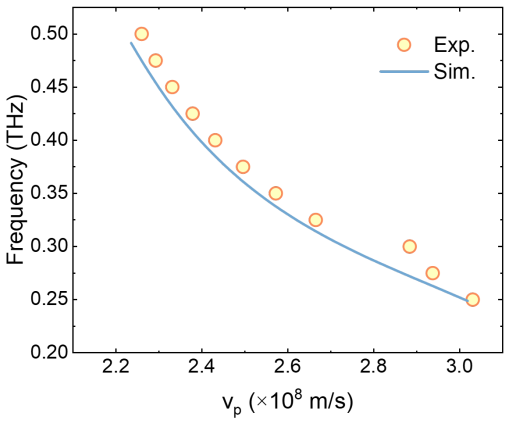

Fig. S8. The experimental and simulative phase velocities of the DVM. The results suggest that higher (lower) frequencies correspond to slower (faster) phase velocities.

Section 7. Dynamic distributions along path BB.

Near-field characterization along the BB axis of the PCF cross-section reveals time-domain signal distributions (Fig. S9a). Subsequent STSF analysis resolves frequency-dependent temporal-spatial dynamics, as exemplified by mode evolution at 0.3 THz as mapped in Fig. S9b.

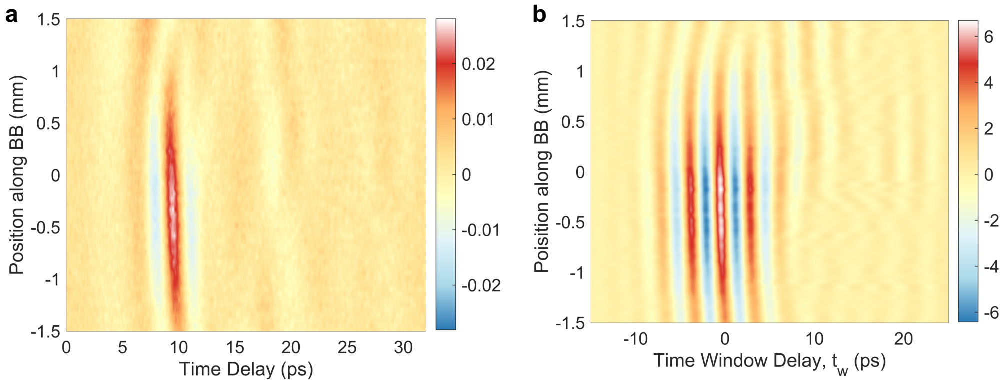

Fig. S9. a, Measured time-domain signal distribution along path BB. **b,** Dynamic frequency component at 0.3 THz along path BB.

Section 8. Electric field patterns of the DVM.

As demonstrated in Fig. 3f, near-field modal profiles of the DVM at distinct frequencies are obtained (Fig. S10). The observed progressive energy localization toward the fiber core with increasing frequency (0.2-0.5 THz) directly correlates to the effective area

reduction trend as quantified in Fig. 4c.

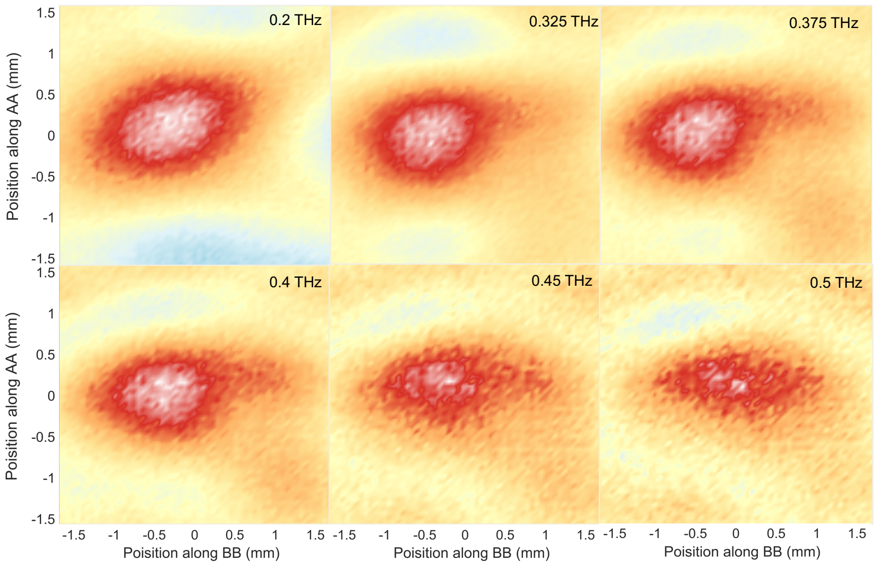

Fig. S10. Measured spatial profiles of the DVM at various frequencies.

Section 9. Measured DVM spatial profiles for 2-cm PCF and parameter extraction.

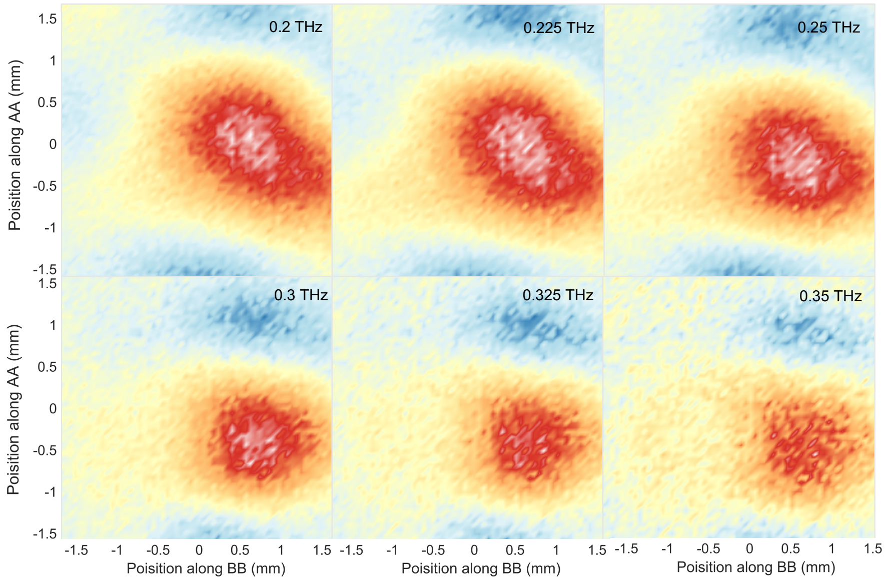

Fig. S11. Measured spatial profiles of the DVM at various frequencies for the PCF length of 2cm.

The experimental spatial profiles of the DVM for a 2 cm-long fiber are shown in Fig. S11. These measurements confirm that the transmitted field remains strongly concentrated at the fiber center, in agreement with the simulated localization of the DVM mode along the propagation direction.

Total propagation loss (α_{tol}) was determined through differential amplitude analysis of electric field (E) measured with two fibers of distinct lengths (L_1 = 1 cm, L_2 = 2 cm). Assuming identical coupling attenuation and negligible nonlinear effects, the loss coefficient is derived from the Beer-Lambert law:

$$\alpha_{tol} = \frac{1}{L_2 - L_1} \ln\left(\frac{E_{max}(L_1)}{E_{max}(L_2)}\right) \qquad (S4)$$

where $E_{\max}(L)$ denotes the peak electric field amplitude at length L. This approach effectively eliminates the influence of coupling-related losses by leveraging the linear proportionality between transmitted amplitude and propagation distance in the weak coupling regime.

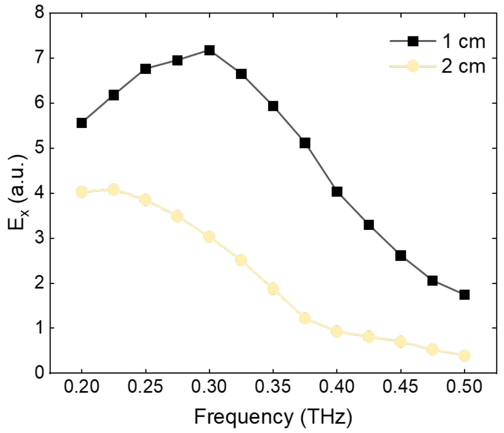

Fig. S12. Measured maximal electric field amplitudes of the DVM for the two fibers of different lengths at different frequencies.

Notably, the accuracy of this confinement loss is influenced by signal-to-noise ratio (SNR) of the DVM. We analyze the SNR of the strongest near-field time-domain signal after propagation through the 1-cm PCF (Fig. 4a bottom panel in the main text), obtaining the frequency-dependent SNR results shown in Fig. S13a. To quantify the confinement loss, we measured the near-field time-domain THz signals transmitted through PCFs of 1-cm and 2-cm lengths (ΔL = 1 cm). The comparison of these two signals allows the confinement-related attenuation to be isolated from systematic

absorption. We then analyzed the SNR of the strongest near-field time-domain signal after propagation through the 1-cm PCF, obtaining the frequency-dependent SNR results shown in Fig. S13a. To successfully detect the confinement loss, the THz signal intensity after an additional propagation distance ΔL must remain above the average noise level, in other words, the SNR after 1 cm propagation must exceed the attenuation incurred by material loss over the additional ΔL, as illustrated in Fig. S13b. The relation refers to as:

$$SNR > e^{2\alpha_{mat}\Delta L}$$

The material-induced attenuation over ΔL = 1 cm is estimated around 7.071 dB at 0.4 THz, while the measured SNR after the initial 1-cm propagation is 3.684 dB. The strong absorption by material thus leads to the observed spectral fluctuations beyond 0.4 THz, where the signal amplitude after extra 1-cm propagation falls below the noise threshold. This forms the reason why unstable measurable data appear in the mode amplitude.

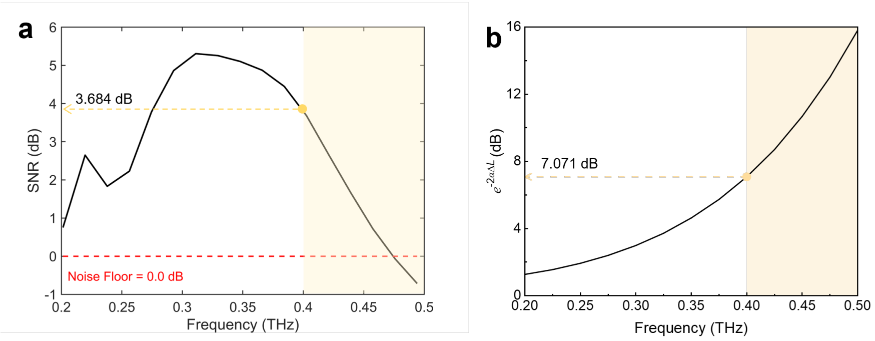

Fig. S13. Comparison between SNR and material loss. a, Frequency-dependent SNR of THz signals after propagation through the 1-cm PCF. **b**, Frequency-dependent attenuation by material absorption over the additional ΔL (1 cm).

Section 10. Effective refractive index of the DVM.

The real and imaginary parts of the effective refractive index were retrieved according to $n_{eff} = k_z/k_0$, as shown in Fig. S14. Material absorption loss can be calculated by $\alpha_{mat}(cm^{-1}) = 4\pi Im(n_{eff})/\lambda_0$, where λ_0 is the wavelength in free space. α_{mat}, dominated by the imaginary part of the effective refractive index, was subtracted to

obtain the confinement loss by $\alpha_{con} = \alpha_{tol} - \alpha_{mat}$.

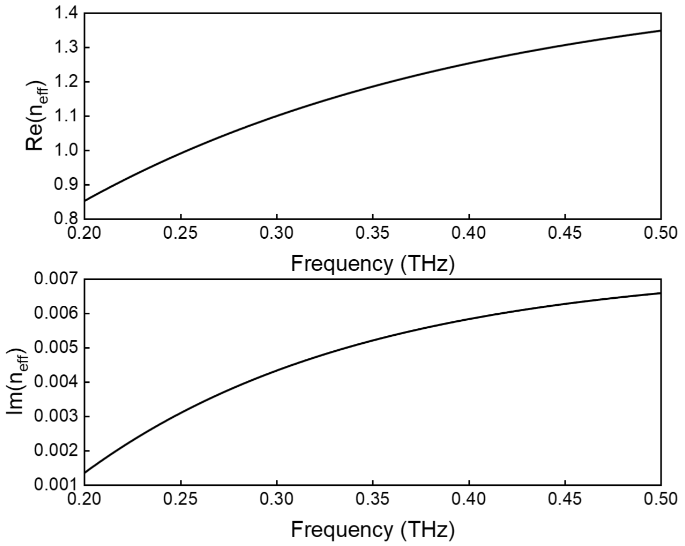

Fig. S14. Real (bottom) and imaginary part (top) of effective refractive index.

Section 11. Zero confinement loss for the DVM in lossless materials.

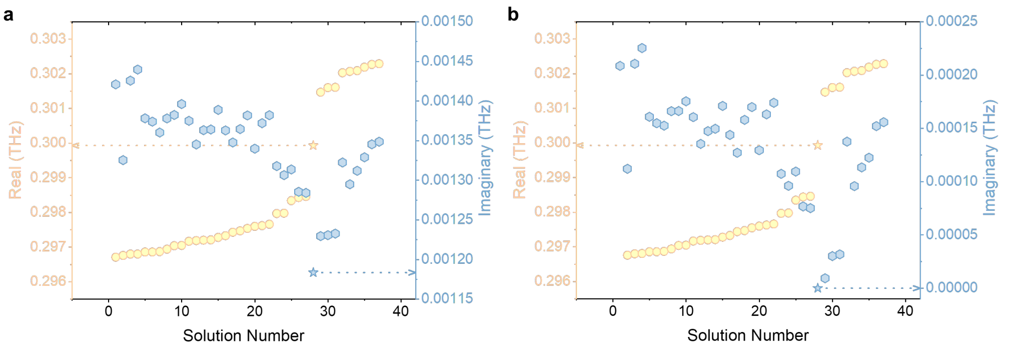

Fig. S15. The eigenfrequencies of the PCF at $k_z d/2\pi$ = 2. a, The refractive index of the host material is set to 1.631 +i 0.009. **b**, The refractive index of the host material is set to 1.631.

Fig. S15a presents numerical eigenfrequency simulations at $k_z d/2\pi = 2$, plotting the real and imaginary components of the eigenfrequencies, with the DVM marked by asterisks. Under finite material absorption, the DVM exhibits imaginary frequency components comparable to those of bulk modes with a finite value. However, when the material loss is eliminated (Im[n] = 0), the imaginary component of the DVM vanishes, as shown in Fig. S15b, while bulk modes retain a finite dissipation. This result confirms that

material absorption is the primary contributor to propagation loss in the SPSM PCF, and that the DVM approaches a near-zero confinement loss under ideal lossless conditions.

Section 12. Schematic diagram of experimental setup.

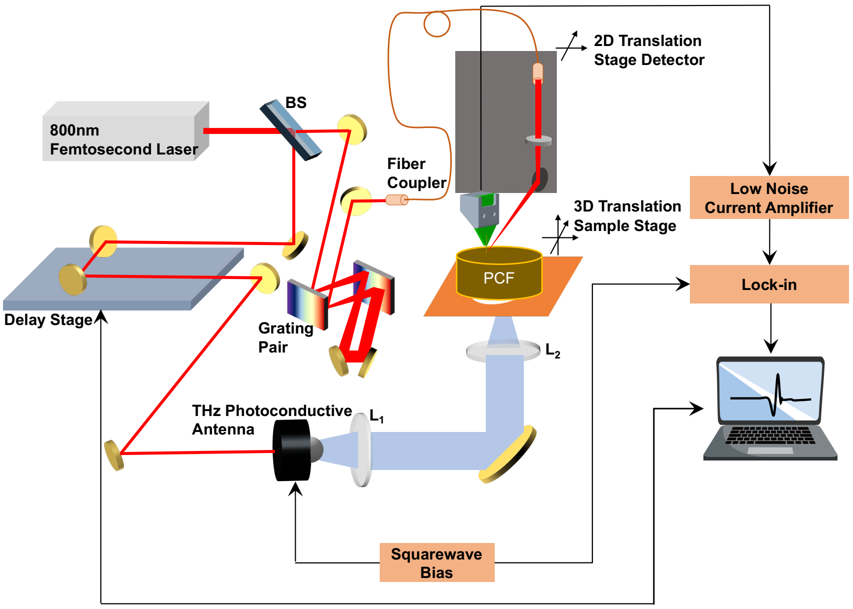

Fig. S16. Schematic diagram of experimental setup. BS: beam splitting. L_1: THz lens (f = 50 mm). L_2: THz lens (f = 35 mm).

Section 13. In-plane bandgap width determined by R_0 and ΔR.

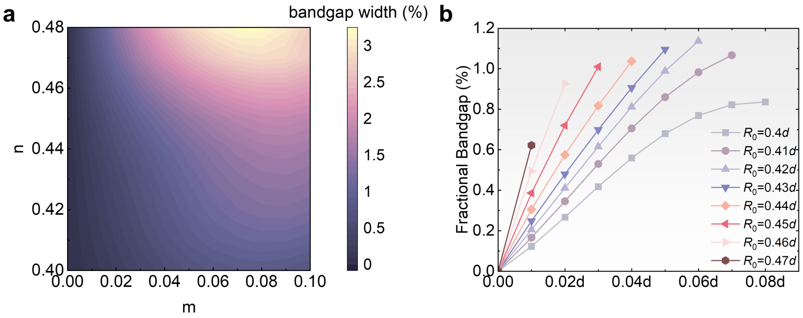

Fig. S17. In-plane bandgap width. a, Bandgap width determined by R_0 and ΔR. **b,** Feasible parameter under the limitation to fabrication accuracy.

In our design, the in-plane bandgap is jointly determined by the original air-hole radius (R_0 = n·d) and the modulation amplitude (ΔR = m·d). To clarify this point, we have

included additional simulation results in Fig. S17a. As can be observed, both R_0 and ΔR strongly affect the bandgap width: larger values of R_0 and ΔR result in a broader in-plane gap. However, practical considerations impose important constraints. If ΔR is too large, adjacent air holes may overlap, leading to structural instability. Moreover, fabrication limitations, particularly the resolution of 3D printing in the THz regime, place strict bounds on how finely such modulations can be realized.

To address these constraints systematically, we extracted the range of feasible parameters, as shown in Fig. S17b. From this set, we selected $R_0 = 0.46d$ and $\Delta R = 0.02d$. This choice achieves a sufficiently wide in-plane bandgap while ensuring structural integrity and manufacturability. In addition, we considered the modal distribution of the DVM with respect to the THz near-field testing system. Specifically, we ensured that the bandgap overlaps with the frequency range where the excitation spectrum of the measurement setup is strongest, thereby optimizing experimental detectability.

Section 14. DVM in *xz* and *yz* planes.

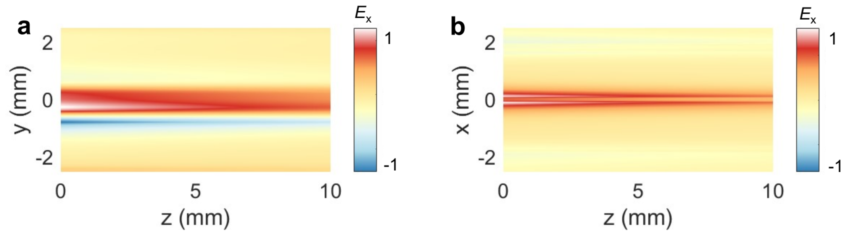

Fig. S18. DVM distributions in the internal PCF. a, E_x field in the *yz* plane at 0.3 THz. **b,** E_x field in the *xz* plane at 0.3 THz.

The simulations of the electric field component (E_x) in the *xz* and *yz* planes of the fiber are presented in Fig. S18. The results clearly demonstrate that the field remains localized around the fiber center. This localization arises because the DVM does not possess an in-plane wavevector; however, due to the intrinsic material loss in the fiber, the field exhibits a gradual attenuation along the propagation direction. This decay is consistent with the expected dissipative behavior of THz waveguides and does not compromise the defect-localized guiding mechanism itself.

Section 15. Transition from localized mode to propagating mode.

We present a more general theoretical framework that explains the transition from a localized mode to a propagating mode upon introducing a non-zero k_z. In the absence of a vertical wave vector ($k_z = 0$), the DVM is a localized mode that is confined spatially within a resonant region, typically described by a transverse field distribution $E(x, y)$. The mode satisfies the Helmholtz equation:

$$\nabla^2 E(x,y) + k^2 E(x,y) = 0,$$

where $k=2\pi/\lambda$ is the wavenumber in the transverse plane. The solution to this equation is a standing wave confined in the transverse plane, and no energy propagates along the fiber axis, i.e., in the z-direction.

When a non-zero vertical wave vector $k_z \neq 0$ is introduced, the DVM transforms from a localized mode to a propagating mode. This can be understood by extending the solution of the wave equation to three dimensions:

$$\nabla^2 E(x,y,z) + k^2 E(x,y,z) = 0,$$

where the total wave vector is now $k^2 = k_x^2 + k_y^2 + k_z^2$, and the mode becomes a superposition of both transverse and longitudinal components. The field distribution in the longitudinal direction can now be expressed as:

$$E(x,y,z) = E(x,y)e^{-ik_z z},$$

where $E(x, y)$ is the transverse component of the field, and $e^{-ik_z z}$ represents the propagation along the fiber axis with the wave vector k_z. The non-zero k_z component introduces propagation, transforming the localized mode into a guided wave.

The transition from a localized to a propagating mode can be understood in terms of the coupled mode theory. When a mode with $k_z \neq 0$ is coupled to a waveguide, the field distribution in the longitudinal direction can no longer remain strictly confined. Instead, the field extends along the fiber axis, resulting in the mode propagating as a waveguide mode.

This mechanism is not unique to our specific fiber design but is a more general phenomenon in photonic systems.

Section 16. Robustness of the DVM.

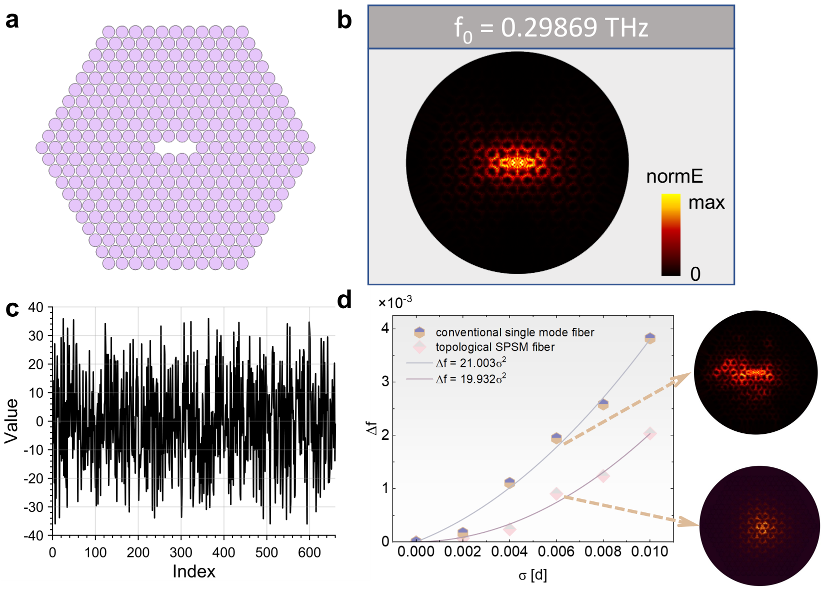

Fig. S19. Robustness comparison between our PCF and conventional PCF. a, Structure of a conventional single-mode THz PCF without topologically protected robustness. b, Operational mode for the single-mode fiber. c, Random function has a mean of zero and a standard deviation of 0.01*d*. d, Frequency shift dependent of various standard deviations for the conventional single-mode fiber and topological SPSM fiber.

First of all, we conduct a robustness comparison with conventional single-mode THz fiber. A single-mode PCF without topological protection is designed, as shown in Fig. S19a. The anisotropy of this fiber results in a single mode localized at the fiber core, which we then contrasted with our topological design, as shown in Fig. S19b. We explored the effect of random perturbations in the position of air holes as defects in both fiber types. The random function defining the position offsets has a mean of zero but allows for varying standard deviations (σ), and we present the effect of a standard deviation of 0.01*d* in Fig. S19c. Our simulations reveal that, as the standard deviation increases, the frequency shift ($\Delta f = (f - f_0)/f_0$, where f_0 is the original frequency under no perturbations) and mode distortion in the conventional fiber and our PCF grow

parabolically, with the conventional fiber exhibiting a significantly larger shift and more pronounced mode deformation compared to the DVM in the topological fiber, as depicted in Fig. S19d.

Furthermore, we investigated the robustness of the DVM against radius defects in the air holes, which are more directly related to the Kekulé modulation in the fiber's design. While position defects (displacement) in the air holes can maintain the DVM up to a standard deviation of $0.01d$, significant changes in the air hole radius (e.g., defects of $0.002d$) lead to the disappearance of the DVM. This behavior is driven by the topological phase transition, which is inherently sensitive to the changes in the air hole radius. As shown in Fig. S20, larger-scale variations in radius compromise the topological protection, resulting in the loss of the DVM.

	0.002σ	0.004σ	0.006σ	0.008σ	0.01σ	0.012σ
position	√	√	√	√	√	×
radius	√	×	×	×	×	×

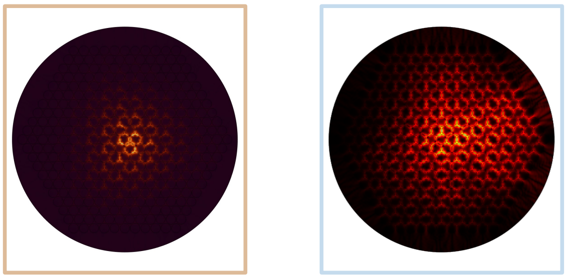

Fig. S20. Robustness of the DVM under position defects and radius defects.

Additionally, we examined the effect of missing air holes on the fiber's mode confinement. As demonstrated in Fig. S21, when air holes near the center of the fiber are missing, the electric field exhibits a defect mode in that region. However, even with such imperfections, the overall DVM remains largely intact, further illustrating the robustness of the topological mode against localized defects.

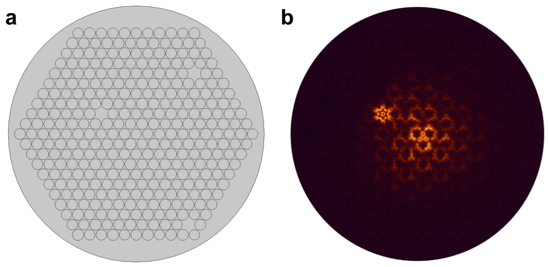

Fig. S21. Robustness of the DVM under missing the air holes.

Section 17. Robustness of the DVM under discrete and broken Kekulé modulations.

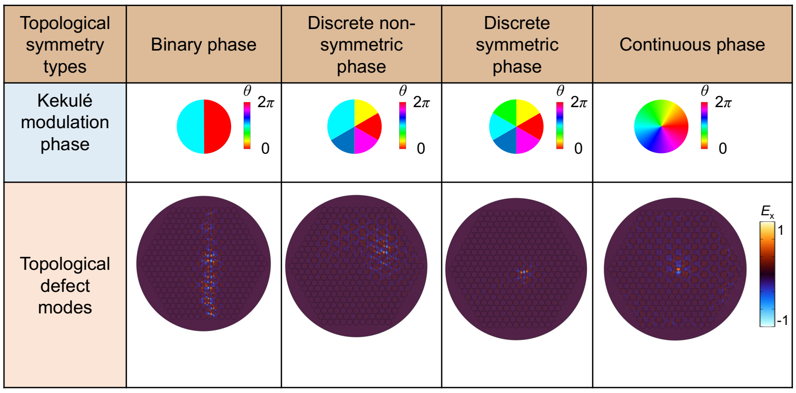

Fig. S22. DVM under different topological symmetry types.

In contrast to the continuous modulation phase distribution analyzed in the main text, where the phase of each air hole is determined directly by its spatial coordinate, we considered two additional cases: (i) binary phase modulation consisting of 0 and π phases, and (ii) discrete phase modulation where each air hole is assigned a fixed phase within a certain range. The binary phase system corresponds to alternating domains with distinct topological indices. In this scenario, the topological state emerges at the domain boundary, manifesting as a boundary-localized mode rather than a vortex-centered defect mode. For the discrete phase case, we designed both "symmetric-phase" structures, where trivial and nontrivial regions are uniformly distributed, and "non-symmetric-phase" structures, where the distribution of trivial and nontrivial regions is unbalanced. The results show that in the "non-symmetric-phase" case, the defect mode is not strictly confined to the fiber center due to the absence of a complete topological

phase transition. By contrast, in the "symmetric-phase" configuration, the DVM remains localized near the center because topological phase transitions occur consistently in all directions, although the confinement is weaker compared with the ideal continuous phase design.

These observations confirm that the existence and robustness of the DVM are indeed sensitive to the details of the protecting symmetry. While continuous-phase modulation offers the strongest localization and robustness, binary and discrete modulations still support topological modes, but with reduced confinement or a shift in their spatial characteristics.

Section 18. Coupling loss of the PCF.

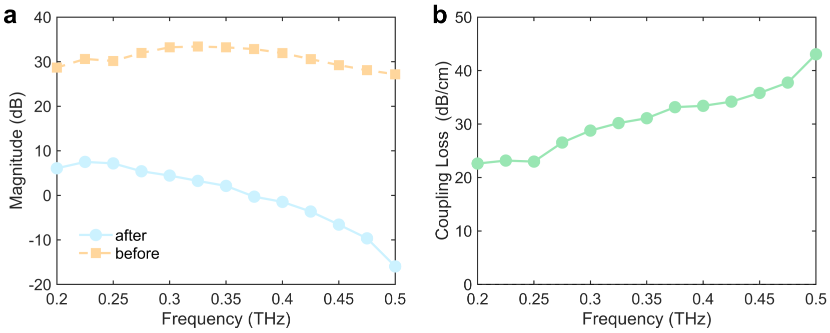

Fig. S23. Coupling loss of the PCF incurred by wavevector mismatch to free space. a, Fourier transforms for the THz time signal before and after coupling the PCF. **b**, Coupling loss derived from the difference in spectral intensity.

The coupling loss in fiber-based THz waveguiding systems is determined by the efficiency with which the input THz beam is coupled into the fiber mode. In our experiments, the coupling efficiency is quantified by comparing the time-domain signals before and after coupling the THz beam into the fiber. As shown in Fig. 4a of the manuscript, we used Fourier transforms of the time-domain signal to assess the spectral strength in the frequency domain (Fig. S23a), with the difference in spectral intensity directly providing the coupling loss, as illustrated in Fig. S23b. From the results, we observe that the coupling loss of the PCF is notably high, with a loss of 30 dB/cm at 0.3 THz. The low coupling efficiency arises because only THz beam within

the frequency range and incidence angles that support SPSM operation, that is, under dispersion-matching conditions, can effectively couple into the mode. As a result, significant coupling loss occurs when coupling the THz beam directly from free space into the fiber mode.

Section 19. Measured dispersion of high-temperature resin in the THz regime

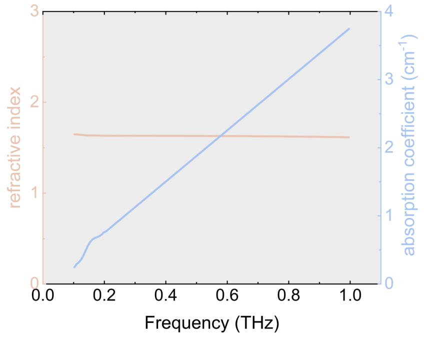

Fig. S24. Measured dispersion of high-temperature resin in the THz regime.

Through THz-TDS measurements, the dispersion of high-temperature resin was measured, yielding the refractive index and absorption coefficient shown in Fig. S24. The refractive index is 1.631 in the range of 0.2–0.5 THz, while the absorption coefficient (α_{resin}) varies linearly with frequency. From $\alpha_{\text{resin}} = (4\pi k/c)f$, the extinction coefficient k = 0.009 is calculated, and the complex refractive index of the high-temperature resin is $n = 1.631 + i0.009$.

Section 20. Polarization alignment-free characteristic of the DVM

As shown in Fig. 5 of the main text, the in-plane electric field of the topological mode exhibits a vortex-like polarization distribution, characterized by an azimuthally varying polarization direction and a continuous 2π phase winding around the fiber center. This field configuration can be expressed in cylindrical coordinates as

$$\mathbf{E}(r,\varphi) = E_r(r)\hat{r} + E_\varphi(r)e^{il\varphi}\hat{\varphi}$$

where l denotes the topological charge (in our case $l = 1$). The phase term $e^{il\varphi}$ signifies that the polarization vector rotates continuously with the azimuthal angle. Due to this

azimuthally symmetric vortex polarization, the mode does not favor a specific in-plane direction of excitation. In other words, any linearly polarized incident field can be decomposed into a superposition of the orthogonal components that match the local polarization basis of the vortex mode:

$$\mathbf{E}_{inc} = E_0(\hat{x}\cos\theta + \hat{y}\sin\theta) = \frac{E_0}{\sqrt{2}}(\hat{e}_+ e^{-i\theta} + \hat{e}_- e^{i\theta})$$

where $\hat{e}_\pm$ are the circular polarization basis vectors. Because the vortex mode couples to both spin components equivalently through the rotationally symmetric field distribution, coupling efficiency remains independent of polarization angle θ. As a result, the excitation of the topological vortex mode is effectively polarization-alignment-free.

Section 21. The topological modes under distinct winding numbers

As illustrated in Fig. S25, we analyzed the cases of $|w|$ = 1, 2, 3 within the bandgap. The results show that the magnitude of $|w|$ directly determines the number of topological defect modes formed inside the gap: for $|w|$ = 1, 2, 3, there appear one, two, and three defect vortex states, respectively. These modes emerge as a result of $|w|$-order topological phase transitions.

Specifically, for $|w|$ = 1, 2, 3, the corresponding point-group symmetries of the structures are C_3, D_3, and C_3, respectively. Based on group-theoretical analysis, the irreducible representations of these symmetry groups reveal that for w = 1and w = 3, there are no degeneracies since these groups lack two-dimensional irreducible representations. In contrast, for w = 2, both non-degenerate and degenerate modes appear, corresponding respectively to one-dimensional and two-dimensional irreducible representations of the D_3 group.

The sign of the winding number w determines which sublattice the mode field localizes on: positive and negative winding numbers lead to field distributions localized on opposite sublattices of the photonic crystal lattice.

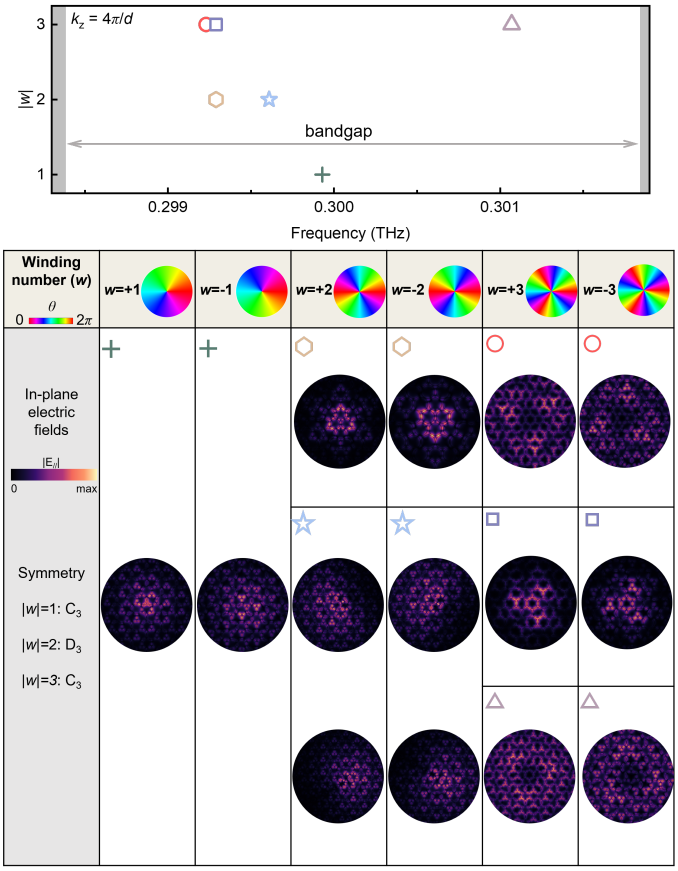

Fig. S25. DVM field patterns with different winding numbers.
